# A cholera outbreak caused by drinking contaminated river water, Bulambuli District, Eastern Uganda, March 2016

**DOI:** 10.1186/s12879-019-4036-x

**Published:** 2019-06-11

**Authors:** Paul Edward Okello, Lilian Bulage, Alex Ario Riolexus, Daniel Kadobera, Benon Kwesiga, Henry Kajumbula, Muhamed Mulongo, Eunice Jennifer Namboozo, Godfrey Pimundu, Isaac Ssewanyana, Charles Kiyaga, Steven Aisu, Bao-Ping Zhu

**Affiliations:** 1 Uganda Public Health Fellowship Program, Kampala, Uganda; 2 Makerere University School of Medicine, Kampala, Uganda; 3 Central Public Health Laboratories, Kampala, Uganda; 4 Bulambuli District Health Office, Bulambuli, Uganda; 5 US Centres for Disease Control and Prevention, Atlanta, USA

**Keywords:** Cholera, Outbreak, Uganda

## Abstract

**Background:**

A cholera outbreak started on 29 February in Bwikhonge Sub-county, Bulambuli District in Eastern Uganda. Local public health authorities implemented initial control measures. However, in late March, cases sharply increased in Bwikhonge Sub-county. We investigated the outbreak to determine its scope and mode of transmission, and to inform control measures.

**Methods:**

We defined a suspected case as sudden onset of watery diarrhea from 1 March 2016 onwards in a resident of Bulambuli District. A confirmed case was a suspected case with positive stool culture for *V. cholerae*. We conducted descriptive epidemiologic analysis of the cases to inform the hypothesis on mode of transmission. To test the hypothesis, we conducted a case-control study involving 100 suspected case-patients and 100 asymptomatic controls, individually-matched by residence village and age. We collected seven water samples for laboratory testing.

**Results:**

We identified 108 suspected cases (attack rate: 1.3%, 108/8404), including 7 confirmed cases. The case-control study revealed that 78% (78/100) of case-patients compared with 51% (51/100) of control-persons usually collected drinking water from the nearby Cheptui River (OR_MH_ = 7.8, 95% CI = 2.7–22); conversely, 35% (35/100) of case-patients compared with 54% (54/100) of control-persons usually collected drinking water from borehole pumps (OR_MH_ = 0.31, 95% CI = 0.13–0.65). The index case in Bwikhonge Sub-county had onset on 29 February but the outbreak had been on-going in the neighbouring sub-counties in the previous 3 months. *V. cholera* was isolated in 2 of the 7 river water samples collected from different locations.

**Conclusions:**

We concluded that this cholera outbreak was caused by drinking contaminated water from Cheptui River. We recommended boiling and/or treating drinking water, improved sanitation, distribution of chlorine tablets to the affected villages, and as a long-term solution, construction of more borehole pumps. After implementing preventive measures, the number of cases declined and completely stopped after 6th April.

## Background

Cholera is a severe and acute diarrheal disease caused by *Vibrio cholerae*. It is an epidemic-prone disease and has caused seven pandemics historically since 1817 [1, 2]. *V. cholerae* is an non-invasive organism, affecting the small intestine through secretion of the cholera toxin targeting the intestinal mucosal epithelium, leading to the characteristic acute watery diarrhoea [3]. Two sero-groups of *V. cholerae*, 01 and 0139, cause outbreaks [1]. Sero-group 01 causes the majority of the outbreaks worldwide, while 0139 is more common in Southeast Asia [1]. Cholera is transmitted to humans through consumption of water or food contaminated with *V. cholerae* [3]. Cholera outbreaks often occur in poor communities with limited access to clean drinking water and proper sanitation [4–6]. Globally, two thirds of the estimated 2.8 million annual cholera cases and 88% of the 91,000 annual fatalities occur in Sub-Saharan Africa [7].

If left untreated, a cholera patient can develop severe dehydration within an hour of the onset of symptoms and may die within 2–3 h [1]. The case-fatality rate of untreated cholera can be as high as 50–60% [1]. With adequate rehydration therapy, the case fatality rate (CFR) can be reduced to < 1% [1, 8]. The C is often higher in children and the elderly [1]. In Uganda, the CFR has declined over time due to improved clinical management, from 4 to 7% during the outbreaks in the late 1990s to about 2–3% during the recent outbreaks [9].

In late January 2016, a cholera outbreak started in the southern part of Bulambuli District, Eastern Uganda, near the border with Kenya. Combined control efforts by the Uganda Ministry of Health and other non-governmental organizations kept the outbreak at a low level but did not completely stop it. In late March, a sharp increase in cholera cases occurred in Bwikhonge Sub-county of the district. We investigated this outbreak to determine its scope and mode of transmission, and to inform evidence-based interventions.

## Methods

### Study site

We focused the investigations in Bwikhonge Sub-county (N01 ° 24, 259′, E 034°20.540′) which had the bulk of the suspected case-persons. Approximately 1/3 of the residents in Bwikhonge Sub-county reside in Bwikhonge Parish around Cheptui River. There is also a swamp in the sub-county. Cheptui River, a few bore hole pumps and swamp water have been the alternative sources of drinking water for the residents.

### Case definition

We defined a suspected case as sudden onset of watery diarrhea from 01st March 2016 to 9 April 2016 in a resident (aged ≥5 years) of Bwikhonge Sub-county, Bulambuli District. A confirmed case was a suspected case with positive culture for *V. cholerae* from a stool sample.

### Case finding

We reviewed in-patient records and line-list at the only cholera treatment centre in Bwikhonge Sub-county. We conducted active case finding by visiting all parishes in Bwikhonge Sub-county with the help of members of community health workers, and updated the line-list.

### Hypothesis generation

Using a standardized case investigation form, we interviewed 40 case-patients conveniently found at the cholera treatment centre and the community about their histories of food and water intake and practices during the possible exposure period. Persons interviewed were all residents of Bwikhonge Sub-county. We also analyzed the line-list data by time, place, and person to generate hypotheses on the mode of transmission.

### Case-control study

We conducted a case-control study in which we interviewed 100 case-patients and 100 asymptomatic controls. Cases were identified from the suspected case-persons in the line-list. We used pair matching to select controls, in which one randomly selected control was paired to a case by age-group and village of residence at a ratio of 1:1 [10]. Cases and controls were randomly selected from different households. If a household had more than one case-patient, we only selected the one with the earliest onset to participate in the case-control study. We used a structured questionnaire to collect information on water-intake history and practices among the case- and control-persons.

### Statistical methods

We computed attack rates by sex, age, and parish of residence using population data from the national census and data provided by the Uganda Bureau of Statistics [11]. We computed the proportions of persons who used the various water sources during the cholera outbreak period. In the case control study, we used StatCalc in Epi Info 7.2.2.2 to determine the proportions of cases and controls: considering a power of 90%, two-sided confidence level of 95%, a case-control ratio of 1:1 with 30% of cases exposed and 10% of controls exposed, we would require about 85 cases and 85 controls; we then took 100 cases and 100 controls.

We measured the associations between the drinking water exposure sources and cholera illness using the Mantel-Haenszel method to estimate odds ratios (OR) and their confidence intervals, accounting for pair-matching of cases and controls. Bore hole pump water was taken to be the reference source since it tested relatively safer given the negative bacterial culture, but also because it is regarded to be a clean water source [12].

### Laboratory investigation

We cultured 13 stool samples collected from suspected cholera case-persons to confirm the cholera diagnosis. We also collected 7 water samples from different water sources within the affected communities (including 2 from borehole pumps, 1 from a swamp, and 4 from Cheptui River) for bacteriological culture identification of *V. cholerae*. We collected water specimens from Cheptui River 1 meter from the edge of riverbank and from just below the surface, in pre-sterilized one-litre autoclavable glass bottles. We collected 2 l of river water from each of the four water-collection points along the river which were most frequently used by local residents. We collected 2 l of borehole pump water from each of the two borehole pumps, and 2 l of swamp water. We transported the water samples in cool boxes to the laboratory and processed within 6 h of collection.

We used a standard procedure to culture for *V. cholera* from the river water specimen. A pad was made from sterile gauze, placed into a clean funnel, and 2 l of water specimen was filtered through the gauze. The gauze with debris was immersed into 125 mls of double strength alkaline peptone water in sterile screw cap glass conical flasks and incubated at 37 °C for 18–24 h. The procedure was repeated for all other water samples, taking care to avoid cross-contamination between samples by changing funnels and employing aseptic techniques. Using a 10 μl wire loop, a loop full of the enriched culture was picked from just beneath the surface of the broth and streaked onto thiosulfate-citrate-bile salts-sucrose (TCBS) agar and incubated aerobically at 37 °C for 18–24 h. The gauze pad was left suspended in the alkaline peptone water for at least 2–5 days for possible repeat analyses. Presumptive colonies (yellow, shiny colonies, 2–4 mm in diameter) were identified using standard procedures [3, 9]. We tested the *V.cholera* isolates for antimicrobial susceptibility using modified Kirby-Bauer disk diffusion method.

### Environment assessment

We assessed hygiene conditions at sources of drinking water and water for household use. We assessed human activities likely to result into river water contamination along the Cheptui River and around the few bore hole water pumps. We also assessed human waste disposal practices in the communities.

## Results

### Descriptive epidemiology

We identified 108 cases in Bwikhonge Sub-county (overall attack rate: 1.3%). Of the 13 stool specimens taken from case-persons, 7 yield *V. cholerae* by culture. Two elderly persons (> 60 years) died (case-fatality rate: 1.9, 2/108). The outbreak affected male and female residents equally in all age-groups, suggesting that the exposure that led to this outbreak was ubiquitous. Among the five parishes in the sub-county, Bwikhonge had the highest attack rate at 3.1% (Table 1).

**Table 1:** Cholera attack rates by parish in Bwikhonge sub-county in Bulambuli district, March 2017

Category	Cases (*n* = 108)	Population (*n* = 8,404)	Attack rate (%)
Sex			
Male	53	4042	1.3
Female	55	4362	1.3
Age (years)			
5-12	20	1790	1.1
13-19	6	1437	0.41
20-30	30	1890	1.6
31-59	31	1681	1.8
60-90	21	345	6.1
Parish			
Bwikhonge	83	2689	3.1
Bulumera	23	1849	1.2
Bunalwere	1	1597	0.063
Buwabwala	1	756	0.13
Buwekanda	0	1513	0

The attack rates were similar in males and females, all age groups were affected with the highest attack rate occuring in the elderly persons. Bwikhonge subcounty had the highest attack rate at 3 persons per 100

**Fig. 1:**
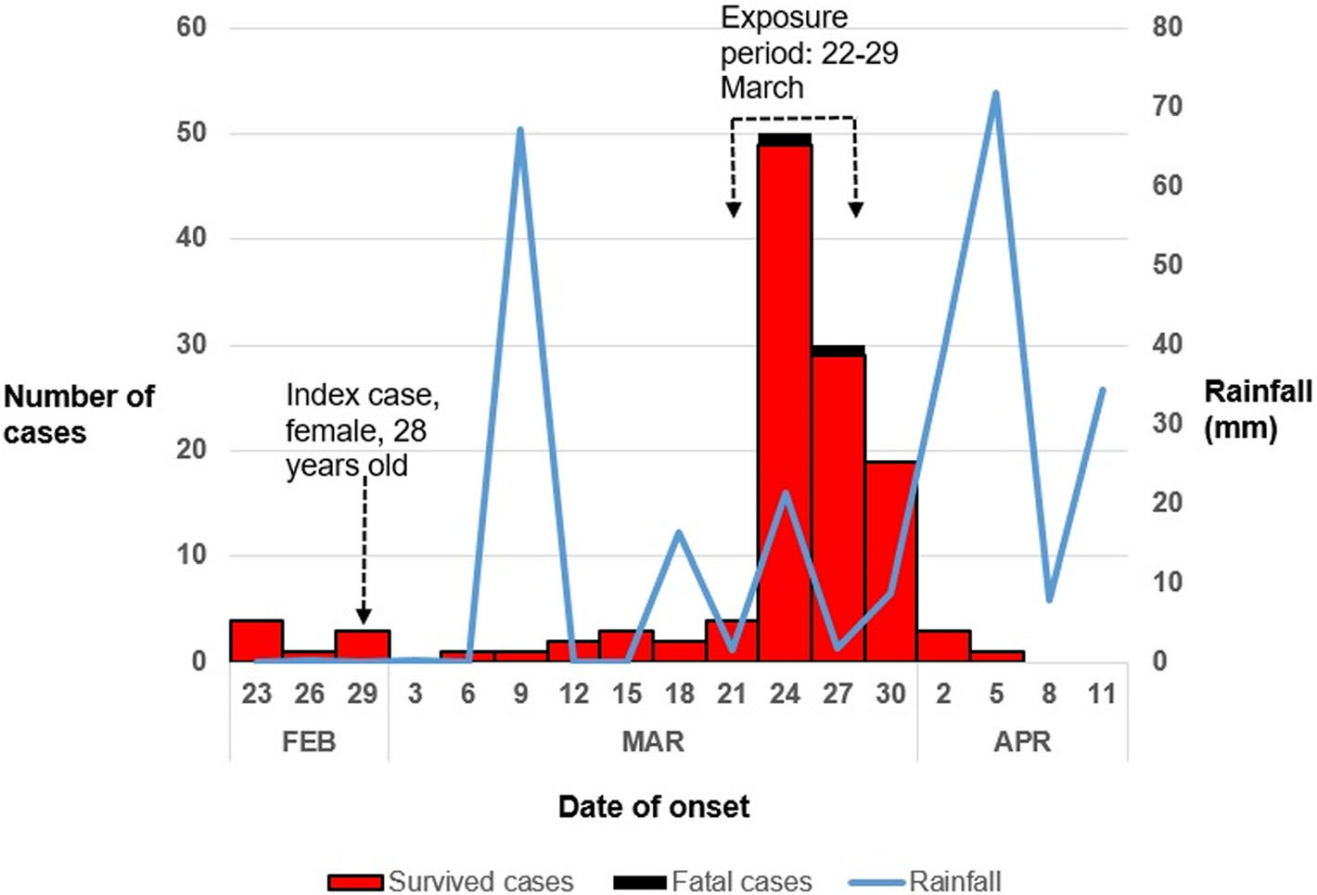
Epidemic curve of cholera outbreak in Bwikhonge sub-county in Bulambuli District, March 2016

We could not establish the role of the index case-person in the spread the infection to the other case-persons in the sub-county. On 24 March, the number of cases increased steeply and peaked on 25 March in the presence of moderate rainfall 20-30 mm per day. Afterwards the cases gradually decreased but remained elevated for about a week (Fig. 1). This epidemic curve is consistent with a continuous common source outbreak [10]. Based on the incubation period for cholera (a few hours to 5 days), the exposure that caused this outbreak likely occurred on 22 March and lasted about a week.

The two fatal cases had onsets on 24 and on 27 March. A rainfall peak on 9th March occurred about 2 weeks before the spike in the number of cases on 23rd March and therefore could not be directly linked to the rise in the number of cholera cases. The hypothesis-generating interviews showed that, of the 40 case-persons interviewed, (67%) reported using Cheptui river as the source of domestic utility water for washing kitchen utensils and clothes, 76% reported having used unboiled or untreated Cheptui river water for drinking during the exposure period, 95% were neither treating nor boiling the water before drinking, 24% did not have latrines and open defecation was observed in 24% of the case households. All cases reported having been taking hot food during and after the exposure period and this largely rules out the possibility of food borne cholera and other food borne diarrhoeal diseases.

Based on the findings from the descriptive epidemiology and the hypothesis-generating interviews, we hypothesized that water in Cheptui River was the probable source of the cholera outbreak and that drinking unboiled or untreated river water caused the outbreak.

### Case-control study

In the case-control study the characteristics of cases were generally similar to that of controls; we compared the levels of education, occupation, and human waste disposal practices**.** There were no obvious occupational association with the cholera outbreak (Table 2**)**.

**Table 2:** Case control study: characteristics of cholera cases (*N* = 100) and controls (*N* = 100)

Variable	Case n (%)	Control n (%)
Level of education of household head		
None	14 (14)	18 (18)
Primary	65 (65)	61 (61)
Secondary	21 (21)	21 (21)
Level of education of case/control		
None	9 (9.2)	16 (16)
Primary school	69 (70.4)	65 (65)
Secondary school	20 (20.4)	19 (19)
Occupation		
Peasant farmer	80 (80)	77 (78)
Soldier/police officer	1 (1)	1 (1)
Pupil/student	19 (19)	22 (22)
Latrine availability		
Available	68 (69.4)	75 (76)
Not available	30 (30.6)	24 (24)
Evidence of open defecation		
Yes	24 (25)	10 (10)
No	72 (75)	86 (90)

There was no notable difference between cases and controls in terms of level of education, and occupation. However, 31% of the cases did not have latrines compared to 24% for controls. Open defecation was observed among 25% of the case’s households compared to 10% for controls. Although latrine availability and open defecation do not directly cause cholera transmission, they point to hygiene problems in the community

From the case-control study, we identified the key risk factors for cholera transmission in this investigation by stratified analysis using the Mantel Haenszel method. People who used untreated borehole pump water for drinking were 70% protected from cholera. (OR_M-H_ = 0.31, 95% CI = 0.13–0.65). On the contrary, people who used untreated Cheptui river water for drinking were up to 8 times as likely to get cholera (OR_M-H_ = 7.8, 95% CI = 2.7–22). Drinking swamp water (which we saw appeared turbid) was not statistically associated with cholera acquisition (OR_M-H_ = 2.5, 95% CI = 0.8–8.0). The use of Cheptui river water and swamp water for household utility was also significantly associated with cholera disease (Table 3).

**Table 3:** Case-control study: risk factors for cholera due to water utility or drinking water from various water sources (*n* = 100 Cases, *n* = 100 controls)

Variable	Cases n (%)	Controls n (%)	OR_M-H_^a^	95%CI
Untreated borehole water for drinking				
Yes	35(36)	54(54)	0.31	0.13-0.65
No	63(64)	46(46)	ref	
Untreated Cheptui river water for drinking				
Yes	76(78)	54(51)	7.8	2.7-22.0
No	22(22)	49(49)	ref	
Untreated swamp water for drinking				
Yes	11(11)	5(5)	2.5	0.80-8.0
No	87(89)	95(95)	ref	
Untreated borehole water for domestic utility				
Yes	35(36)	40(40)	0.70	0.30-1.5
No	63(64)	60(60)	ref	
Untreated Cheptui river water for domestic utility				
Yes	81(83)	69(69)	3.8	1.4-10.0
No	17(17)	31(31)	ref	
Untreated swamp water for domestic utility				
Yes	12(12)	3(3)	5.5	1.2-25.0
No	86(88)	97(97)	ref	

^a^*ORM*_*-H*_ odds ratio of association was computed by stratification or Mantel-Haenszel method. Drinking untreated borehole water was protective, OR_M-H_ **=** 0.31, 95% CI=0.13-0.65) and this result might be due to confounding factors in which persons may have used bore hole water with other water sources in various combinations. Water was drunk or used untreated (and unboiled) from all sources. People who drank untreated Cheptui river water were up to 8 times as likely to have had cholera compared to those who did not (OR_M-H_ =7.8, 95% CI = 2.7-22.0) and this relatively larger OR_M-H_ value supports the water borne hypothesis. People who used untreated Cheptui river water for routine work were up to 4 times as likely to have had cholera compared to those who did not (OR_M-H_ =3.8, 95% CI = 1.4-13.0)

### Laboratory and environmental investigations

Subtyping of *V.cholera* isolated from stool specimens of the 7 confirmed case-persons indicated that all had *V. cholerae* (O1 Ogawa). In addition, 2 of the 7 water samples obtained from the frequently used water-collection points also yielded *V. cholerae* (01 Ogawa) by culture; one was from Cheptui River and the other was from a swamp. Two bore hole water samples tested negative for *V.cholera* by culture. The other three water samples that tested negative were collected from two other bore hole pumps and a point along Cheptui river. Antimicrobial susceptibility tests showed that the *V. cholerae* 01 Ogawa that caused the outbreak was susceptible to tetracycline, ciprofloxacin, chloramphenicol, and cotrimoxazole but resistant to ampicillin.

We visited the local Cheptui River shores on several occasions and witnessed some of the local people collecting visibly dirty river water in portable containers to take to their homes. Some of the case-persons admitted using the river water for drinking after boiling or treating, but also use it for other domestic purposes. We saw five functional bore holes pumps in use and located in hygienic environments. The bore holes were sources of visibly clean water that the locals drank directly, but some of the locals detested it for its slightly salty taste, preferring the river water that they reported to be more palatable. The swamp water that some of the residents used for drinking or household utility appeared unclean and turbid. A few of the local shops sold water purification tablets that the locals found expensive to use continuously, and the community health workers reported that sometimes the tablets were distributed in the villages free of charge by governmental and non-governmental organisations. Families reported that they could not afford the cooking fuel (firewood or charcoal) necessary for boiling water daily so as to ensure safe drinking water. Community health workers reported that diarrhoeal diseases were common in the sub-county, but cholera had not occurred before. We noted that open defeacation was common around homesteads in the densely populated Bwikhonge Parish. The problem of human faecal deposits was also common along the banks of Cheptui River. The practice of washing dirty clothes near the river banks was also common, with waste laundry water disposed off in ways that flows back to the river. Local leaders explained that many families lack pit latrines because the local soil structure does not sustain the construction of intact pit latrines without pit walls collapsing after only a few of days following construction. It rained heavily on 2 of the 6 days of the outbreak investigation (up to 70 mm rainfall per day between 02nd and 06th April), and we witnessed surface rain water draining into the river Cheptui at various places.

## Discussion

In this investigation, we found out that the most likely cause of the cholera outbreak was the practice of people directly consuming contaminated water from River Cheptui. This is evidenced by the fact that the highest odds of contracting cholera is linked to the group of persons who directly drank untreated river water from the stratified analysis. The bore hole water that we saw appeared clean and was recommended by the local health workers for direct drinking, in preference to all other local water sources. Where available, deep groundwater sources (including bore hole pumps) and/or gravity-flow supplies from springs are generally preferable and safer, as they require less treatment [13]. The five boreholes in this sub-county appeared to be insufficient for a population of 8404 people and therefore some people were forced to use river water for drinking in its contaminated state as well as for other domestic purposes. In comparison, Cholera epidemics elsewhere in Africa have been linked to drinking from or bathing in lakes [14], drinking contaminated river water [15], eating at large funeral feasts [16], and eating cold leftover foods [15]. Outbreaks may be seasonal, with seasonality associated with environmental parameters such as rainfall and temperature [17]. The Bwikhonge outbreak occurred in March at the beginning of the rainy season.

Although we were able to determine the likely exposure period for the major peak, we could not identify what actually happened during this period. The index case in Bwikhonge sub-county was a 28 year old woman who was admitted in the cholera treatment centre on 29 February the very day of the onset and before the onset of persistent rain, but could not be reached for interview to establish her role in the outbreak. We considered heavy rains as a possible culprit, but the last heavy rains (about 65 mm) in the area had occurred on 9th March, about 2 weeks earlier than the peak in number of cases on 23rd March; the 2 weeks is way beyond the incubation period of cholera. However, the peak of the outbreak occurred during rainy days when rainfall measured between 15 and 30 mm per day. The outbreak had been on-going in the neighbouring southern sub-counties in the previous 3 months.

Environmental assessments revealed common open defecation along the banks of River Cheptui. The most affected villages and neighbourhoods lay around the neighbourhood of River Cheptui. The culture positive test for *Vibrio cholera* at some water points is confirmatory of the presence of the aetiological agent of cholera in Cheptui River water at the time of the investigation. The outbreak was possibly sustained by the presence of *V.cholerae* in water at common water collection points. The contaminants were sustained through other means including direct faecal contamination and washing of soiled clothes of cholera victims at common water collection points along the river; these activities were evident during the investigation. Human faeces were commonly seen in the open around some homesteads and at several places near the banks of Cheptui River, adding further evidence of possible contaminants of water sources by dirty rainwater since the outbreak occurred at the beginning of the rainy season in March. Latrine coverage in the cholera outbreak area stood at 58% according to the district statistics, this forced people without latrines to defecate in the open, followed by the washing of the excreta downslope by rain water to unprotected or open water sources including the nearby Cheptui River. Contamination of Cheptui river water sources meant that a wide variety of people using the river water were affected as seen in the attack rates. According to the local people, Cheptui River as the only river traversing the sub-county had not flooded in the recent year to cause heavy contamination with faecal matter. The locals also reported that Cholera outbreaks had previously occurred in the neighbourhood of this region in previous years; in such outbreaks, river networks in the region were suspected to have been affected and these communities habitually drink river water directly without any treatment. The hydrology of the Cheptui River comprises the fact that it is one of the many rivers that form a radial network of rivers on the slopes of the Elgon mountain ecosystem on both the Uganda and Kenya sides of the Elgon slopes [18]. The river reportedly flows all year round, with no reported drought or flooding affecting it [18]. Cheptui River and other rivers of the Mt. Elgon ecosystem provide food, water, recreational services, and act as buffers during periods of low precipitation and / or long dry seasons [18]. The river is also utilized by locals for several socio-economic activities including agriculture, small-scale industries, tourism, settlements, and wild-life conservation [18]. The human activities around the river makes it prone to contamination with pathogenic or chemical agents. The CFR of 1.9% in the cholera outbreak in Bulambuli District occurred in a setting in which there were already multiple efforts by the district health team and other partners to contain the outbreak. The CFR is high taking the Latin American experience as a model; mortality rates for cholera epidemics should be kept below 1% according to the World Health Organization [1, 19]. The reported CFR for cholera in Uganda decreased from 4 to 7% in the late 1990’s to about 2–3% during 2004 to 2010 [9, 20] indicating improvements in preparedness and management of cholera outbreaks across the entire country. A well-organized disease outbreak response in a country with a well-established diarrhea disease control program can limit the CFR to less than 1% [20, 21]. Other factors have been put forward as possibly contributing to high CFR above 1%, including bacterial virulence factors, poor nutrition, and poor immunity of infected persons, delays in diagnosis, and difficulties of accessing appropriate treatment [22]. All of these factors may have been at play to various levels in the incidence we investigated.

The cholera treatment centre that treated the victims during the outbreak used doxycycline, tetracycline, ciprofloxacin and erythromycin as alternatives. Severe cholera cases in adults were treated with a combination of intravenous rehydration and doxycycline 300 mg single dose or with ciprofloxacin 1 g single dose or tetracycline 500 mg every 6 h for 3 days. Severe cholera cases in children under 12 years were treated with intravenous rehydration with erythromycin 25-50 mg/kg every 6 h for 3 days or doxycycline 2 mg/kg single dose (if > 8 years old) or ciprofloxacin 20 mg/kg single dose. Children above 12 years with severe cholera received doxycycline 2 mg/kg single dose or ciprofloxacin 20 mg/kg 12 hourly for 3 days. Regional resistance of *V.cholerae* to ampicillin was first reported in a survey [23] in 1997 in Kenya, Tanzania and Rwanda and so our finding might be a problem of an older origin.

In the short term, the government of Uganda should equip the people in rural areas with the knowledge that treating or boiling water before drinking prevents cholera and other diarrhoeal diseases, and thereafter provide sustainable means of domestic water purification. In the long term, the government should ensure expanded coverage of borehole water pumps and of treated and piped water supply, improved sanitation, and hygiene promotion across all parts of the country to prevent cholera outbreaks. The key intervention that controls a cholera outbreak is one that blocks the transmission of the aetiological agent soon after the passing of cholera faeces [1]. Ultimately, good sanitation is the key to the control of cholera. Nevertheless the aquatic environment continues to serve as a perpetual reservoir given its vastness and the adaptability of the *V. cholerae* to survive under such conditions for extended periods [1].

Our investigation had some limitations: molecular typing for cholera toxin gene was not done since some biotypes of Ogawa serotype are non-toxigenic [24]. However, the case definitions were met to the satisfaction of our objectives. The monitoring of antimicrobial resistance patterns was not done periodically during the course of the outbreak in the region to monitor the development of resistance. In the large cholera outbreak in Southern Africa, resistance was found to develop within 3 months for some drugs [4].

## Conclusions

This was a continuous common source outbreak caused by drinking contaminated water from Cheptui River. By the time the investigation was completed, the number of cases had been reduced to one or none per day after concerted efforts by the district health team. To the community leaders we emphasized boiling and treating drinking water, restriction on washing clothes near drinking water collection points, use of latrines and avoidance of open defecation near water sources in the short term. The long term solution included construction of more boreholes in Bwikhonge Sub-county, expansion of treated and piped water supply in the district, and improvement in human waste disposal system through use of well-constructed latrines and where possible modern flush toilets. Our recommendations were in agreement with the on-going efforts by the district health team.
